# Protein-to-genome alignment with miniprot

**DOI:** 10.1093/bioinformatics/btad014

**Published:** 2023-01-17

**Authors:** Heng Li

**Affiliations:** Department of Data Sciences, Dana-Farber Cancer Institute, Boston, MA 02215, USA; Department of Biomedical Informatics, Harvard Medical School, Boston, MA 02115, USA

## Abstract

**Motivation:**

Protein-to-genome alignment is critical to annotating genes in non-model organisms. While there are a few tools for this purpose, all of them were developed over 10 years ago and did not incorporate the latest advances in alignment algorithms. They are inefficient and could not keep up with the rapid production of new genomes and quickly growing protein databases.

**Results:**

Here, we describe miniprot, a new aligner for mapping protein sequences to a complete genome. Miniprot integrates recent techniques such as k-mer sketch and vectorized dynamic programming. It is tens of times faster than existing tools while achieving comparable accuracy on real data.

**Availability and implementation:**

https://github.com/lh3/miniport.

## 1 Introduction

Sequencing technologies have been rapidly evolving in recent years. The advent of long-read sequencing, especially accurate long-read sequencing (Wenger *et al.*, 2019), has enabled high-quality genome assembly at scale (Cheng *et al.*, 2021, 2022; Nurk *et al.*, 2020). After we sequence and assemble the genome of a new species, the immediate next step is to annotate genes.

There are three ways to annotate gene structures: *ab initio* gene prediction, aligning RNA-seq data from the same species and mapping known genes with cross-species alignment. While *ab initio* gene prediction works well for bacterial genomes, it is error-prone for Eukaryotic genomes that may contain large introns. In a recent benchmark (Scalzitti *et al.*, 2020), all the evaluated gene finders miss ∼50% nucleotides in annotated exons and predict ∼50% extra sequences not in exons. If we have RNA-seq data, we can map short or long RNA-seq reads (Dobin *et al.*, 2013; Li, 2018) and reconstruct transcripts from the alignment (Kovaka *et al.*, 2019). This will give much more accurate gene structures than *ab initio* gene prediction. Unfortunately, RNA sequencing adds extra cost and may miss genes lowly expressed in the tissues being sequenced. We still rely on cross-species alignment to derive a complete gene set and to transfer known functional annotations to the new genome.

For very closely related genomes, we can reconstruct gene structures from whole-genome alignment (Fiddes *et al.*, 2018) or from the alignment of gene regions (Shumate and Salzberg, 2020). These methods would not work well for genomes at longer evolutionary distances because intron sequences are less conserved and this will affect the quality of the alignment. Aligning the more conserved coding regions (Gotoh, 2008; Li *et al.*, 2007) may alleviate the issue. However, for distantly related species, even coding nucleotide sequences are not conserved well. Just as we almost exclusively use protein sequences to reconstruct the phylogeny of distant homologs, Ensembl (Aken *et al.*, 2016) and other gene annotation pipelines (Haas *et al.*, 2008; Holt and Yandell, 2011; Brůna *et al.*, 2021) also heavily rely on protein-to-genome alignment especially when the annotation of closely related species is not available.

There are several protein-to-genome aligners that pinpoint exact splice sites: GeneWise (Birney and Durbin, 1997; Birney *et al.*, 2004), Exonerate (Slater and Birney, 2005), GeneSeqer (Usuka and Brendel, 2000), GenomeThreader (Gremme *et al.*, 2005), genBlastG (She *et al.*, 2011), ProSplign (Kapustin *et al.*, 2008) and Spaln2 (Gotoh, 2008; Iwata and Gotoh, 2012). They all take a protein and a nucleotide sequence as input and output spliced alignment between them. GeMoMa (Keilwagen *et al.*, 2019) additionally requires gene structures in the source genome as input. It aligns exons without splicing and then connects exon alignments. As gene structures are conserved across species, this strategy simplifies alignment and potentially reduces spurious hits but it would not easily work for proteins from a variety of species.

Among the tools above, only Spaln2, GenomeThreader and GeMoMa are practical for whole-genome alignment. They can align several hundred proteins per CPU hour and may take a couple of days to align a few hundred thousand proteins often needed to annotate a genome without closely homology. Protein-to-genome alignment is time consuming.

It is challenging to develop a fast and accurate protein-to-genome alignment algorithm. The core of such alignment is a dynamic programming (DP) that jointly considers affine gap penalties, introns and frameshift. It is perhaps the most complex DP for pairwise alignment. In addition, as we will show later, a successful aligner functions like a gene finder and has to properly model splice signals, which is not a trivial task, either. On top of these, we need to fit these complex methods to an efficient implementation with modern computing techniques. This is partly why we have over a hundred short-read mappers (Alser *et al.*, 2021) but only three protein-to-genome mappers capable of whole-genome alignment.

In this article, we will describe miniprot, a new protein-to-genome aligner developed from scratch. We will demonstrate its performance and accuracy on real data along with the few existing algorithms.

## 2 Materials and methods

Miniprot broadly follows the seed-chain-extend strategy used by minimap2 (Li, 2018). It indexes the genome k-mers in all six open reading frames (ORFs) on both strands. During alignment, miniprot extracts k-mers on a query protein, finds seed anchors and then performs chaining. It closes unaligned regions between anchors and extends from terminal anchors with DP.

### 2.1 Notations of strings

For a string *T*, let |*T*| be its length and *T*[*i*], i=1,…,|T|, be the *i*th symbol in *T*. *T*[*i, j*], 1≤i≤j≤|T|, is the substring starting at *i* and ending at *j* inclusively. In this article, *T* denotes the genome sequence over the nucleotide alphabet and *P* denotes the protein sequence over the amino acid alphabet.

### 2.2 Reduced alphabet

There are 20 amino acids. We need at least five bits to encode each amino acid. To encode protein sequences more compactly, we reduce the amino acid alphabet using the SE-B(14) scheme by Edgar (2004), except that we merge N and D. More exactly, we map amino acid groups to integers as follows: A→0, ST→1, RK→2, H→3, ND→4, EQ→5, C→6, P→7, G→8, IV→10, LM→11, FY→12, W→13, ∗→14 and X→15, where ∗ denotes the stop codon and X denotes an amino acid.

Under this encoding, if two amino acid groups only differ at the lowest bit (e.g. group ‘A’ and ‘ST’), the two groups tend to be similar. We may flip the lowest bit of an integer to generate more seeds and thus to increase the seeding sensitivity. We did not use this strategy as miniprot seems reasonably sensitive to real data.

### 2.3 Indexing the genome

Miniprot only indexes a subset of k-mers in the genome. Suppose ϕ(a) maps an amino acid *a* to a 4-bit integer with the scheme described above. The integer encoding of a *k*-long protein sequence *P* can be recursively defined as ϕ(P)=ϕ(P[1,k−1])×16+ϕ(P[k]). ϕ(P) has 4*k* bits. Let B=ψ(ϕ(P)) where ψ(·) is an invertible integer hash function (Li, 2016) over $\left[0,2^{4k}\right)$. Then, *B* is also an integer with 4*k* bits. By default, miniprot only indexes *B* if the lowest bit of *B* is 0. We thus sample 50% of k-mers in average with a high-quality hash function ψ(·).

Internally, miniprot treats each genome sequence and its reverse complement as two independent sequences. It enumerates all ORFs of 30 amino acids or longer and samples 6-mers from translated ORFs with the strategy above. For each selected k-mer *R* at position *x*, miniprot stores (ψ(ϕ(R)),⌊x/256⌋) in a hash table with the key being ψ(ϕ(R)) and the value being an array of positions. We do not retain the base resolution at the indexing step such that we can use 32-bit integers to store positions for a genome up to $2^{39}$ (= 2^32^ × 256/2) base pairs in size. Without binning, miniprot would have to use 64-bit integers to store positions in a human genome, which would double the index size.

### 2.4 Chaining

The miniprot chaining algorithm is similar to the minimap2 algorithm. However, because the miniprot index does not keep the exact genome positions, the gap size calculation needs to be modified. For completeness, we will describe the full chaining equation here.

Let 2-tuple (x,y) denote a seed match, also known as an anchor, between binned position *x* on the genome and residue position *y* on the protein. Suppose $\left(x_{i},y_{i}\right)$ and $\left(x_{j},y_{j}\right)$ are two anchors with $x_{i}\leq x_{j}$ and $y_{i}<y_{j}$. The minimum possible gap size between the two anchors, in the unit of base pair, can be calculated by

(1) $$g(i,j)=\{\begin{array}{cc} 3\Delta y-256(\Delta x-1) & \text{if}\;3\Delta y<256(\Delta x-1)\\ 3\Delta y-256(\Delta x+1) & \text{if}\;3\Delta y>256(\Delta x+1)\\ 0 & \text{otherwise} \end{array}$$

with $\Delta x=x_{j}-x_{i}$ and $\Delta y=y_{j}-y_{i}$. When g(i,j)=0, we do not know if there is a gap due to binning. Meanwhile, g(i,j)>0 indicates a definitive insertion to the genome and g(i,j)<0 indicates a definitive deletion.

Given a list of anchors sorted by genomic position *x*, let f(j) be the maximal chaining score up to the *j*th anchor in the list. f(j) can be calculated with

(2) $$\begin{array}{c} f(j)=\max\{\underset{1\leq i<j}{\max}\{f(i)+\alpha (i,j)-\gamma (g(i,j))\},k\} \end{array}$$

where *k* is the k-mer length (six amino acids by default), g(i,j) is calculated by Equation (1) and $\alpha (i,j)=\min\{y_{j}-y_{i},k\}$ is the number of matching residues between the anchors. The gap penalty function γ(·) is

(3) $$\gamma (g)=\{\begin{array}{cc} 0 & \text{if}\;g=0\\ \infty & \text{if}\;|g|\geq G\\ g/3+\beta {\;\text{log}\;}_{2}(g+1) & \text{if}\;0<g<G\\ \min\{|g|/3,\beta {\;\text{log}\;}_{2}(|g|+1)\} & \text{if}-G<g<0 \end{array}$$

Here, *G* is the maximum intron size (200 kb by default) and β is the weight of the logarithm gap penalty (0.75 by default). The logarithm term allows miniprot to join exons over introns.

After the initial round of chaining for each protein, miniprot selects the top 30 chains and performs another round of chaining in local regions around these top chains. In the second round, miniprot indexes all 5-mers on both the protein and the genome subsequences without binning. This finds better chains and retains the base resolution of each anchor. Miniprot uses $g^{\prime }(i,j)=3\Delta y-\Delta x$ to compute gap lengths and applies the same gap penalty Equation (3) during chaining.

### 2.5 Residue alignment with DP

Miniprot uses DP to close gaps between anchors in chains and to extend from terminal anchors. The DP aims to find gaps, frameshift and splicing at the same time as is demonstrated as follows (‘Geno’ for the genome sequence, ‘Tran’ for the translated protein sequence in the alignment and ‘Prot’ for the query protein sequence):

**Table:** 

Geno:	GAG	GCC	---	CGC	TCA	CC	gt…ag	CACA	AG	CGC	TAT	A	GCC	TAC
Tran:	E..	A..	---	R..	S..	P.		.T..	$$	R..	Y..	+	A..	Y..
	|	|		|		|		|		|	|		|	|
Prot:	E	A	F	R	-	P		T	E	R	Y		A	Y

In this example, symbol ‘$’ denotes frameshift substitutions and ‘+’ denotes frameshift insertions. We will explain their differences later. In this section, we will first review the AE86 DP formulation for affine gap cost (Altschul and Erickson, 1986) and then derive the DP equation for protein-to-genome alignment.

### 2.6 DP with affine gap cost

Under the affine gap cost, a gap of length *g* costs q+e·g. A direct formulation of the DP looks like

(4) $$\begin{array}{c} \{\begin{array}{ccc} M_{ij} & = & \max\{M_{i-1,j-1},I_{i-1,j-1},D_{i-1,j-1}\}+s(i,j)\\ I_{ij} & = & \max\{M_{i,j-1}-q,I_{i,j-1},D_{i,j-1}-q\}-e\\ D_{ij} & = & \max\{M_{i-1,j}-q,D_{i,j-1}-q,D_{i-1,j}\}-e \end{array} \end{array},$$

where ‘*M*’ represents the matching state, ‘*I*’ the insertion state, ‘*D*’ the deletion state and s(i,j) gives the score between the residue at position *i* on the target sequence and the residue at position *j* on the query. If we define

$$\begin{array}{c} H_{ij}=\max\{M_{ij},I_{ij},D_{ij}\} \end{array}.$$

Equation (4) becomes

(5) $$\begin{array}{c} \{\begin{array}{ccc} I_{ij} & = & \max\{H_{i,j-1}-q,I_{i,j-1}\}-e\\ D_{ij} & = & \max\{H_{i-1,j}-q,D_{i-1,j}\}-e\\ H_{ij} & = & \max\{H_{i-1,j-1}+s(i,j),I_{ij},D_{ij}\} \end{array} \end{array}.$$

Equation (5) is the AE86 formulation. It invokes fewer comparisons. When there are more states, AE86 may save more comparisons and simplify the DP equation.

### 2.7 DP for protein-to-DNA alignment

In a similar manner, we can derive the DP for protein-to-DNA alignment, allowing frameshifts but not splicing:

(6) $$\begin{array}{c} \{\begin{array}{ccc} I_{ij} & = & \max\{H_{i,j-1}-q,I_{i,j-1}\}-e\\ D_{ij} & = & \max\{H_{i-3,j}-q,D_{i-3,j}\}-e\\ H_{ij} & = & \max\{H_{i-3,j}+s(i,j),I_{ij},D_{ij},H_{i-1,j-1}-f,\\ & & H_{i-2,j-1}-f,H_{i-1,j}-f,H_{i-2,j}-f\} \end{array} \end{array}$$

It is similar to Equation (5) except for codon phase transitions with a penalty of *f*. We have two types of frameshift. The first type is created by inserting one or two bases to the DNA sequence (symbol ‘+’ in the example above) and the second type by deleting one or two bases in a codon (‘$’ in the example). These are modeled by the four $H_{\cdot ,\cdot }$ terms on the last line of Equation (6). This equation is broadly similar to Zhang *et al.* (1997).

### 2.8 DP for protein-to-genome alignment

When aligning proteins to genomes, we need to keep phases through introns. We add three additional states, *A*, *B* and *C*, for Phase-0, Phase-1 and Phase-2 introns, respectively. Our final formulation is

(7) $$\begin{array}{c} \{\begin{array}{ccc} I_{ij} & = & \max\{H_{i,j-1}-q,I_{i,j-1}\}-e\\ D_{ij} & = & \max\{H_{i-3,j}-q,D_{i-3,j}\}-e\\ A_{ij} & = & \max\{H_{i-1,j}-r-d(i-1),A_{i-1,j}\}\\ B_{ij} & = & \max\{H_{i-1,j-1}-r-d(i),B_{i-1,j}\}\\ C_{ij} & = & \max\{H_{i-1,j-1}-r-d(i+1),C_{i-1,j}\}\\ H_{ij} & = & \max\{H_{i-3,j}+s(i,j),I_{ij},D_{ij},H_{i-1,j-1}-f,\\ & & H_{i-2,j-1}-f,H_{i-1,j}-f,H_{i-2,j}-f,\\ & & A_{ij}-a(i),B_{ij}-a(i-2),C_{ij}-a(i-1)\} \end{array} \end{array}$$

where *r* is cost of an intron, and d(·) and a(·) model splice signals. The great majority of introns start with GT and end with AG across all species. For a simple model, we may define:

$$\begin{array}{c} d(i)=\{\begin{array}{cc} 0 & \text{if}\;T[i+1,i+2]=\mathrm{GT}\\ p & \text{otherwise} \end{array} \end{array}$$

and

$$\begin{array}{c} a(i)=\{\begin{array}{cc} 0 & \text{if}\;T[i-1,i]=\mathrm{AG}\\ p & \text{otherwise} \end{array} \end{array}.$$

This still allows non-GT-AG splicing but penalizes such introns by cost *p*. We will describe a more sophisticated model in the next section.

It is worth noting that when the DP transitions from state *H* to *B* at position *i*, the Phase-1 intron *B* starts at i+1; when the DP transitions from *B* to *H* at *j*, the intron ends at j−2. The DP ignores the split codon bridging the two exons around the Phase-1 intron. Phase-2 intron state *C* is treated similarly. Not scoring split codons is a weakness of our equation.

Though not explicitly derived from a Hidden Markov model (HMM), Equation (7) is similar to the Viterbi decoding of the 6-state HMM employed by GeneWise (Birney *et al.*, 2004) and Exonerate (Slater and Birney, 2005). To that end, our formulation should have comparable accuracy to the two older aligners if they are parameterized the same way.

We implemented Equation (7) with striped DP (Farrar, 2007). We used 16-bit integers to keep scores and achieved 8-way parallelization for x86_64 CPUs with SSE2 or ARM64 CPUs with the NEON instruction set. Our implementation is over 50 times faster than GeneWise and Exonerate in their exact mode.

### 2.9 Splice models

We observed that under distant homology, the splice model may have a large influence on the junction accuracy, confirming Iwata and Gotoh (2012).

The most common splice pattern in all species is GT-AG with GT at the donor site (5′-end of an intron) and AG at the acceptor site (3′-end of an intron). We occasionally see GC-AG and AT-AC at ∼1% frequency in total (Sheth *et al.*, 2006). Among the GT-AG class, we more often observe GTR-YAG from yeasts to mammals (Irimia and Roy, 2008), where R denotes A or G and Y denotes C or T.

The default miniprot splice model considers the signals above. Using human data from Sibley *et al.* (2016), we estimated that 99.81% of acceptor sites are AG and only 0.10% are AC. In the BLOSUM scaling (Henikoff and Henikoff, 1992), an AC acceptor site would be penalized by $2{\;\text{log}\;}_{2}99.81/0.10\approx 20$. We can adapt this approach for three bases at either the donor or the acceptor sites. In our final model,

$$d(i)=\{\begin{array}{cc} 0 & \text{if}\;T[i+1,i+3]=\mathrm{GTA}\;\text{or}\;\mathrm{GTG}\\ 8 & \text{if}\;T[i+1,i+3]=\mathrm{GTC}\;\text{or}\;\mathrm{GTT}\\ 15 & \text{if}\;T[i+1,i+2]=\mathrm{GC}\\ 21 & \text{if}\;T[i+1,i+2]=\mathrm{AT}\\ 30 & \text{otherwise} \end{array}$$

and

$$\begin{array}{c} a(i)=\{\begin{array}{cc} 0 & \text{if}\;T[i-2,i]=\mathrm{CAG}\;\text{or}\;\mathrm{TAG}\\ 8 & \text{if}\;T[i-2,i]=\mathrm{AAG}\;\text{or}\;\mathrm{GAG}\\ 21 & \text{if}\;T[i-1,i]=\mathrm{AC}\\ 30 & \text{otherwise} \end{array} \end{array}$$

In mammals and even *Drosophila*, the last exon base adjacent to a donor site is more often a G, and we often see a poly-pyrimidine (i.e. C or T) sequence close to an acceptor site. Our human splice model considers these signals. It is also applicable to species with the sequence features above, including *Drosophila*.

Exonerate uses a position-specific weight matrix over ∼10 positions to model splice sites. Spaln2 additionally considers branching sites and provides pre-trained models for a variety of species. Miniprot adopts a relatively simple model with fewer parameters. This makes the model more general but may affect the accuracy of alignment. We are considering a second pass with a splice model trained from the first pass. This strategy is often used in mainstream gene finders (Brůna *et al.*, 2021).

### 2.10 Avoiding pseudogenes

If a spliced gene has an unspliced pseudogene, the unspliced pseudogene may get a better DP score because the alignment to the pseudogene does not pay intron penalties. To reduce the effect of pseudogenes, miniprot recalculates a DP score between the query protein and the translated coding region without introns. In addition, miniprot further penalizes single-exon alignment by intron open score *r* in Equation (7) in case a pseudogene is aligned better by chance.

## 3. Results

### 3.1 Evaluation datasets

To evaluate the accuracy of miniprot, we collected the protein-coding gene annotations of various species: human (*Homo sapiens*) from Gencode v41, mouse (*Mus musculus*) from Gencode M30, zebrafish (*Danio rerio*) and fruit fly (*Drosophila melanogaster*) from Ensembl v107 and mosquito (*Anopheles gambiae*) from Ensembl metazoan v54. We selected the longest protein for each gene to reduce redundant sequences. We mapped zebrafish and mouse proteins to the primary assembly of the human reference genome GRCh38 and mapped mosquito proteins to the Drosophila BDGP6 genome.

### 3.2 Evaluated tools

To evaluate what aligners can map proteins to a whole genome, we randomly sampled 1% of zebrafish proteins and mapped with various aligners. Only miniprot-0.7, Spaln2-2.4.13c (Iwata and Gotoh, 2012), GeMoMa-1.9 (Keilwagen *et al.*, 2019) and GenomeThreader-1.7.3 (Gremme *et al.*, 2005) could finish the alignment in an hour. GenomeThreader found <30% of coding regions in Spaln2 or miniprot alignment. It is not sensitive enough for the human–ish divergence and thus not evaluated on the full dataset.

When running Spaln2, we applied option ‘-Q7 -T# -yS -LS -yB -yZ -yX2’ where ‘#’ specifies the species-specific splice model. Option ‘-LS’ enables local alignment and yields sligtly better alignment overall. Option ‘-yB -yZ -yX2’ apparently has no effect for human–zebrafish alignment but it greatly improves the junction accuracy of the fly–mosquito alignment. We let Spaln2 choose the maximum intron and gene size automatically. Miniprot finds introns up to 200 kb in length by default. We changed this value to 50 kb for fly–mosquito alignment.

We ran GeMoMa with MMseqs2 (Steinegger and Söding, 2017) as the underlying engine. We evaluated the best-unfiltered alignment of each protein as GeMoMa discarded most alignments in the final output. We tried to specify the maximum intron length to 200 kb but GeMoMa took more than 320 GB memory and was killed on our cluster. We thus used 50 kb for all alignment. GeMoMa crashed for the human–mouse dataset at the splice alignment step.

In principle, we could localize a protein with a whole-genome mapper above and then run GeneWise, GeneSeqer and Exonerate in local regions. However, this would not evaluate mapping accuracy. In addition, Iwata and Gotoh (2012) have already shown Spaln2 outperformed these older tools. We thus ignored them in evaluation.

### 3.3 Evaluating protein-to-genome alignment

We aligned zebrafish proteins to GRCh38 using all tools (Table 1). With human-specific splice models, miniprot is slightly more accurate than Spaln2 on most metrics. Nonetheless, for proteins mapped by both miniprot and Spaln2, Spaln2 could find more correct junctions. Looking at proteins Spaln2 aligned better, we observed Spaln2 is more sensitive to small introns and small exons, while miniprot tends to merge them to adjacent alignments. We speculate this may be caused by two factors. First, Spaln2 uses a more sophisticated splice model and may be putting more weight on splice signals than residue alignment. It may create an intron even if the alignment is weak. Second, the Spaln2 developers observed that heuristics may be doing better than strict DP around short introns or exons. In one case, Spaln2 correctly created an exon with one amino acid. Miniprot under the current setting would not produce such an alignment. On the other hand, while Spaln2 found more correct junctions for proteins mapped by both miniprot and Spaln2, it also produced more false junctions related to small exons and introns. It is not clear to us what is the best balance point.

**Table 1.** Evaluating protein-to-genome alignment

Genome species	Human	Human	Human	Human	Human	Human	Human	Fruit fly	Fruit fly	Fruit fly
Protein species	Zebrafish	Zebrafish	Zebrafish	Zebrafish	Zebrafish	Mouse	Mouse	Mosquito	Mosquito	Mosquito
Aligner	Miniprot	Miniprot	Spaln2	Spaln2	GeMoMa	Miniprot	Spaln2	Miniprot	Spaln2	GeMoMa
Splice model	Human	General	Human	Default	N/A	Human	Human	Human	Fruit fly	N/A
Elapsed time (s)	267	257	10 708	11 097	8718	164	3736	34	2528	3378
Peak RAM (GB)	21.8	22.5	9.3	8.9	146.9	15.3	5.6	3.9	2.7	53.5
No. of protein	25 007	25 007	25 007	25 007	25 007	21 844	21 844	13 094	13 094	13 094
No. of multi-exon	16 866	17 104	13 643	13 854	23 109	17 065	16 865	6675	5630	11 420
No. of predicted junc.	157 918	161 295	151 388	209 312	204 764	167 446	171 241	22 614	27 582	43 203
No. of non-ovlp. junc.	482	802	1206	15 658	5712	330	852	488	877	5997
No. of confirmed junc.	145 545	144 734	136 916	129 645	153 781	162 675	162 551	19 774	22 606	25 513
% confirmed junc.	92.16	89.73	90.44	61.94	75.10	97.15	94.93	87.44	81.96	59.05
% base SN	63.11	63.16	57.16	55.74	67.02	90.10	88.62	56.10	50.21	65.08
% base SP	95.43	94.91	95.11	86.75	88.70	97.26	95.27	96.69	97.35	96.10

*Note*: Protein-to-genome alignments are compared to the annotated genes in ‘Genome species’. ‘# multi-exon’ gives the number of proteins mapped with multiple exons. A splice junction (junc.) is confirmed if it is annotated in ‘Genome species’ with exact boundaries; is non-overlapping (non-ovlp.) if the intron in the junction is not overlapping with annotated introns. ‘% confirmed junc.’ is the percentage of predicted junctions that are confirmed. Base sensitivity (base SN) is the fraction of annotated coding regions on the longest transcripts that are covered by alignments. Base specificity (base SP) is the fraction of genomic bases in alignments that are covered by annotated coding regions.

GeMoMa is more sensitive than both miniprot and Spaln2, finding more junctions and more annotated coding regions. It however has lower junction accuracy. We could tune miniprot for increased sensitivity but we decided to keep the current behavior as the additional alignments are less accurate.

For the human–mouse alignment, miniprot is again slightly better than Spaln2. GeMoMa crashed. On the more challenging fly–mosquito dataset, Spaln2 has higher junction sensitivity and higher base specificity than miniprot. GeMoMa continues to have the highest sensitivity but lower junction accuracy and base specificity.

Miniprot is over 30 times faster than Spaln2 and GeMoMa. The performance gap between miniprot and Spaln2 increases with divergence. This is potentially because Spaln2 has to invoke DP through introns more often when it does not see overlapping high-scoring segment pairs (HSPs) and cannot initiate ‘sandwich DP’ (Wu and Watanabe, 2005) to skip introns. With a much faster DP implementation, miniprot can afford to align through all introns regardless of sequence divergence. It thus has more stable performance. Always aligning through introns might be a contributing factor to the higher specificity of miniprot even though Spaln2 has a more careful algorithm.

## 4 Discussions

Miniprot is a fast protein-to-genome aligner comparable to existing tools in accuracy. Its primary use case is to assist gene annotation. At present, the Ensembl pipeline (Aken *et al.*, 2016) still relies on GeneWise (Birney *et al.*, 2004) and Exonerate (Slater and Birney, 2005). MAKER2 (Cantarel *et al.*, 2008; Holt and Yandell, 2011) calls Exonerate. BRAKER2 (Brůna *et al.*, 2021) integrates Spaln2 (Iwata and Gotoh, 2012) and depends on ProtHint (Brůna *et al.*, 2020) which also optionally invokes Spaln2. As older protein-to-genome aligners are relatively inefficient, researchers often use faster approximate methods to localize proteins and then apply these aligners. Now with miniprot, we can perform approximate mapping and exact splice alignment in one go and thus simplify existing pipelines. In addition, when there are closely related species, miniprot could find 90% coding regions in minutes (see ‘base SN’ on the human–mouse dataset in Table 1). It could also be useful for evaluating de novo assemblies (Manni *et al.*, 2021).

Miniprot would not replace full-pledge gene annotation pipelines such as BRAKER2 (Brůna *et al.*, 2021). Miniprot aligns each protein independently. When multiple proteins are mapped to the same locus, miniprot is unable to merge identical gene models or resolve conflicts between alignments. In addition, although miniprot has a realistic splice model, it is not as sophisticated as the BRAKER2 model and is not trained on the target genome. More importantly, BRAKER2 has an *ab initio* gene prediction component and may find genes with weak homology to the input proteins. We are considering to improve our splice model and to develop a separate tool to reconcile overlapping gene models in simple cases. This may provide a convenient annotation pipeline when closely related species are available.

We are evaluating the possibility to support HMMER profiles (Eddy, 2011) as queries. As a HMMER profile summarizes a gene family from multiple species, it may reduce the number of queries and improve the sensitivity of miniprot for distant homologs. There are two algorithmic challenges: seeding and alignment. For seeding, we could generate seeds from the most probable protein or sample multiple seeds directly from the profile; for alignment, we could introduce position-specific substitution cost and gap cost. Nonetheless, the exact solution to these challenges and how much HMMER profiles may improve the alignment remain unknown.

The Vertebrate Genome Project (Rhie *et al.*, 2021), the Darwin Tree of Life project, the Earth Biogenome Project (Lewin *et al.*, 2018) and many other sequencing efforts are going to sequence hundreds of thousands of species to the reference quality in coming years. The annotation of these genomes is as important as the assembly. While we have seen rapid evolution of sequencing technologies and assembly algorithms in recent years, we still heavily rely on core annotation tools developed more than a decade ago. Miniprot is one effort to replace the protein-to-genome alignment step with modern techniques. We look forward to renewed development of other core annotation tools from the community.

## References

[btad014-B1] Aken B.L. et al (2016) The Ensembl gene annotation system. Database (Oxford), 2016, baw093. 27337980 10.1093/database/baw093 PMC4919035

[btad014-B2] Alser M. et al (2021) Technology dictates algorithms: recent developments in read alignment. Genome Biol., 22, 249. 34446078 10.1186/s13059-021-02443-7 PMC8390189

[btad014-B3] Altschul S.F. , Erickson B.W. (1986) Optimal sequence alignment using affine gap costs. Bull. Math. Biol., 48, 603–616. 3580642 10.1007/BF02462326

[btad014-B4] Birney E. , Durbin R. (1997) Dynamite: a flexible code generating language for dynamic programming methods used in sequence comparison. Proc. Int. Conf. Intell. Syst. Mol. Biol., 5, 56–64. 9322016

[btad014-B5] Birney E. et al (2004) Genewise and genomewise. Genome Res., 14, 988–995. 15123596 10.1101/gr.1865504 PMC479130

[btad014-B6] Brůna T. et al (2020) GeneMark-EP+: eukaryotic gene prediction with self-training in the space of genes and proteins. NAR Genom. Bioinform., 2, lqaa026. 32440658 10.1093/nargab/lqaa026 PMC7222226

[btad014-B7] Brůna T. et al (2021) BRAKER2: automatic eukaryotic genome annotation with GeneMark-EP+ and AUGUSTUS supported by a protein database. NAR Genom. Bioinform., 3, lqaa108. 33575650 10.1093/nargab/lqaa108 PMC7787252

[btad014-B8] Cantarel B.L. et al (2008) MAKER: an easy-to-use annotation pipeline designed for emerging model organism genomes. Genome Res., 18, 188–196. 18025269 10.1101/gr.6743907 PMC2134774

[btad014-B9] Cheng H. et al (2021) Haplotype-resolved de novo assembly using phased assembly graphs with hifiasm. Nat. Methods, 18, 170–175. 33526886 10.1038/s41592-020-01056-5 PMC7961889

[btad014-B10] Cheng H. et al (2022) Haplotype-resolved assembly of diploid genomes without parental data. Nat. Biotechnol., 40, 1332–1335. 35332338 10.1038/s41587-022-01261-x PMC9464699

[btad014-B11] Dobin A. et al (2013) STAR: ultrafast universal RNA-seq aligner. Bioinformatics, 29, 15–21. 23104886 10.1093/bioinformatics/bts635 PMC3530905

[btad014-B12] Eddy S.R. (2011) Accelerated profile HMM searches. PLoS Comput. Biol., 7, e1002195. 22039361 10.1371/journal.pcbi.1002195 PMC3197634

[btad014-B13] Edgar R.C. (2004) Local homology recognition and distance measures in linear time using compressed amino acid alphabets. Nucleic Acids Res., 32, 380–385. 14729922 10.1093/nar/gkh180 PMC373290

[btad014-B14] Farrar M. (2007) Striped Smith-Waterman speeds database searches six times over other SIMD implementations. Bioinformatics, 23, 156–161. 17110365 10.1093/bioinformatics/btl582

[btad014-B15] Fiddes I.T. et al (2018) Comparative annotation toolkit (CAT)-simultaneous clade and personal genome annotation. Genome Res., 28, 1029–1038. 29884752 10.1101/gr.233460.117 PMC6028123

[btad014-B16] Gotoh O. (2008) Direct mapping and alignment of protein sequences onto genomic sequence. Bioinformatics, 24, 2438–2444. 18728043 10.1093/bioinformatics/btn460

[btad014-B17] Gremme G. et al (2005) Engineering a software tool for gene structure prediction in higher organisms. Inf. Softw. Technol., 47, 965–978.

[btad014-B18] Haas B.J. et al (2008) Automated eukaryotic gene structure annotation using EVidenceModeler and the program to assemble spliced alignments. Genome Biol., 9, R7. 18190707 10.1186/gb-2008-9-1-r7 PMC2395244

[btad014-B19] Henikoff S. , Henikoff J.G. (1992) Amino acid substitution matrices from protein blocks. Proc. Natl. Acad. Sci. USA, 89, 10915–10919. 1438297 10.1073/pnas.89.22.10915 PMC50453

[btad014-B20] Holt C. , Yandell M. (2011) MAKER2: an annotation pipeline and genome-database management tool for second-generation genome projects. BMC Bioinformatics, 12, 491. 22192575 10.1186/1471-2105-12-491 PMC3280279

[btad014-B21] Irimia M. , Roy S.W. (2008) Evolutionary convergence on highly-conserved 3′ intron structures in intron-poor eukaryotes and insights into the ancestral eukaryotic genome. PLoS Genet., 4, e1000148. 18688272 10.1371/journal.pgen.1000148 PMC2483917

[btad014-B22] Iwata H. , Gotoh O. (2012) Benchmarking spliced alignment programs including Spaln2, an extended version of Spaln that incorporates additional species-specific features. Nucleic Acids Res., 40, e161. 22848105 10.1093/nar/gks708 PMC3488211

[btad014-B23] Kapustin Y. et al (2008) Splign: algorithms for computing spliced alignments with identification of paralogs. Biol. Direct., 3, 20. 18495041 10.1186/1745-6150-3-20 PMC2440734

[btad014-B24] Keilwagen J. et al (2019) GeMoMa: homology-based gene prediction utilizing intron position conservation and RNA-seq data. Methods Mol. Biol., 1962, 161–177. 31020559 10.1007/978-1-4939-9173-0_9

[btad014-B25] Kovaka S. et al (2019) Transcriptome assembly from long-read RNA-seq alignments with StringTie2. Genome Biol., 20, 278. 31842956 10.1186/s13059-019-1910-1 PMC6912988

[btad014-B26] Lewin H.A. et al (2018) Earth BioGenome project: sequencing life for the future of life. Proc. Natl. Acad. Sci. USA, 115, 4325–4333. 29686065 10.1073/pnas.1720115115 PMC5924910

[btad014-B27] Li H. (2016) Minimap and miniasm: fast mapping and de novo assembly for noisy long sequences. Bioinformatics, 32, 2103–2110. 27153593 10.1093/bioinformatics/btw152 PMC4937194

[btad014-B28] Li H. (2018) Minimap2: pairwise alignment for nucleotide sequences. Bioinformatics, 34, 3094–3100. 29750242 10.1093/bioinformatics/bty191 PMC6137996

[btad014-B29] Li H. et al (2007) A cross-species alignment tool (CAT). BMC Bioinformatics, 8, 349. 17880681 10.1186/1471-2105-8-349 PMC2082505

[btad014-B30] Manni M. et al (2021) BUSCO update: novel and streamlined workflows along with broader and deeper phylogenetic coverage for scoring of eukaryotic, prokaryotic, and viral genomes. Mol. Biol. Evol., 38, 4647–4654. 34320186 10.1093/molbev/msab199 PMC8476166

[btad014-B31] Nurk S. et al (2020) HiCanu: accurate assembly of segmental duplications, satellites, and allelic variants from high-fidelity long reads. Genome Res., 30, 1291–1305. 32801147 10.1101/gr.263566.120 PMC7545148

[btad014-B32] Rhie A. et al (2021) Towards complete and error-free genome assemblies of all vertebrate species. Nature, 592, 737–746. 33911273 10.1038/s41586-021-03451-0 PMC8081667

[btad014-B33] Scalzitti N. et al (2020) A benchmark study of ab initio gene prediction methods in diverse eukaryotic organisms. BMC Genomics, 21, 293. 32272892 10.1186/s12864-020-6707-9 PMC7147072

[btad014-B34] She R. et al (2011) genBlastG: using blast searches to build homologous gene models. Bioinformatics, 27, 2141–2143. 21653517 10.1093/bioinformatics/btr342

[btad014-B35] Sheth N. et al (2006) Comprehensive splice-site analysis using comparative genomics. Nucleic Acids Res., 34, 3955–3967. 16914448 10.1093/nar/gkl556 PMC1557818

[btad014-B36] Shumate A. , Salzberg S.L. (2020) Liftoff: accurate mapping of gene annotations. Bioinformatics, 37, 1639–1643. 33320174 10.1093/bioinformatics/btaa1016 PMC8289374

[btad014-B37] Sibley C.R. et al (2016) Lessons from non-canonical splicing. Nat. Rev. Genet., 17, 407–421. 27240813 10.1038/nrg.2016.46 PMC5154377

[btad014-B38] Slater G.S.C. , Birney E. (2005) Automated generation of heuristics for biological sequence comparison. BMC Bioinformatics, 6, 31. 15713233 10.1186/1471-2105-6-31 PMC553969

[btad014-B39] Steinegger M. , Söding J. (2017) MMseqs2 enables sensitive protein sequence searching for the analysis of massive data sets. Nat. Biotechnol., 35, 1026–1028. 29035372 10.1038/nbt.3988

[btad014-B40] Usuka J. , Brendel V. (2000) Gene structure prediction by spliced alignment of genomic DNA with protein sequences: increased accuracy by differential splice site scoring. J. Mol. Biol., 297, 1075–1085. 10764574 10.1006/jmbi.2000.3641

[btad014-B41] Wenger A.M. et al (2019) Accurate circular consensus long-read sequencing improves variant detection and assembly of a human genome. Nat. Biotechnol., 37, 1155–1162. 31406327 10.1038/s41587-019-0217-9 PMC6776680

[btad014-B42] Wu T.D. , Watanabe C.K. (2005) GMAP: a genomic mapping and alignment program for mRNA and EST sequences. Bioinformatics, 21, 1859–1875. 15728110 10.1093/bioinformatics/bti310

[btad014-B43] Zhang Z. et al (1997) Aligning a DNA sequence with a protein sequence. J. Comput. Biol., 4, 339–349. 9278064 10.1089/cmb.1997.4.339

